# Nutraceutical Potential of Astaxanthin in Muscle Metabolism, Exercise Adaptation, and Obesity

**DOI:** 10.3390/nu18010080

**Published:** 2025-12-26

**Authors:** Juliana Silva Siqueira, Serena Castelli, Thiago Luiz Novaga Palacio, Gilda Aiello, Sara Baldelli, Alfonsina D’Amato, Alessandra De Bruno, Mauro Lombardo, Gianluca Tripodi

**Affiliations:** 1 Medical School, São Paulo State University (UNESP), Botucatu 18618-687, Brazil; juliana.siqueira@unesp.br (J.S.S.); thiago.novaga@unesp.br (T.L.N.P.); 2 Department for the Promotion of Human Science and Quality of Life, San Raffaele Open University, Rome, Via di Val Cannuta, 247, 00166 Rome, Italy; serena.castelli@uniroma5.it (S.C.); alessandra.debruno@uniroma5.it (A.D.B.); mauro.lombardo@uniroma5.it (M.L.); gianluca.tripodi@uniroma5.it (G.T.); 3 IRCCS San Raffaele Roma, 00166 Rome, Italy; 4 Department of Pharmaceutical Sciences, University of Milan, Via L. Mangiagalli 25, 20133 Milan, Italy; alfonsina.damato@unimi.it

**Keywords:** astaxanthin, muscle and exercise metabolism, obesity, formulation technologies, nutraceuticals

## Abstract

Astaxanthin (AX), a naturally occurring xanthophyll carotenoid, has attracted growing scientific interest due to its potent antioxidant, anti-inflammatory, and metabolic-regulatory properties. This review provides a critical appraisal of the current evidence regarding the nutraceutical potential of AX in muscle metabolism, exercise adaptation, and obesity management. Preclinical and clinical findings indicate that AX enhances lipid utilization, promotes mitochondrial biogenesis through AMPK activation, and improves endurance and muscle strength, particularly among older adults. Moreover, AX mitigates exercise-induced oxidative stress and muscle damage, thereby supporting recovery and physiological adaptation. In obesity models, AX reduces adipose tissue inflammation, improves insulin sensitivity, and modulates adipokine secretion, suggesting a multifaceted role in metabolic syndrome prevention. Despite robust preclinical data, human trials remain limited and often yield inconsistent outcomes, highlighting the need for well-designed, long-term clinical studies. Emerging evidence highlights the importance of optimized delivery strategies to enhance AX bioavailability and mitochondrial targeting. Nanoemulsions, liposomes, and lipid-based carriers improve stability, absorption, and tissue distribution, thereby potentiating AX’s effects on mitochondrial function and exercise adaptation. Overall, AX emerges as a promising nutraceutical candidate for enhancing muscle function, supporting exercise performance, and managing obesity-related metabolic disease, with delivery innovations representing a critical frontier for future translational applications.

## 1. Introduction

**Figure 1:**
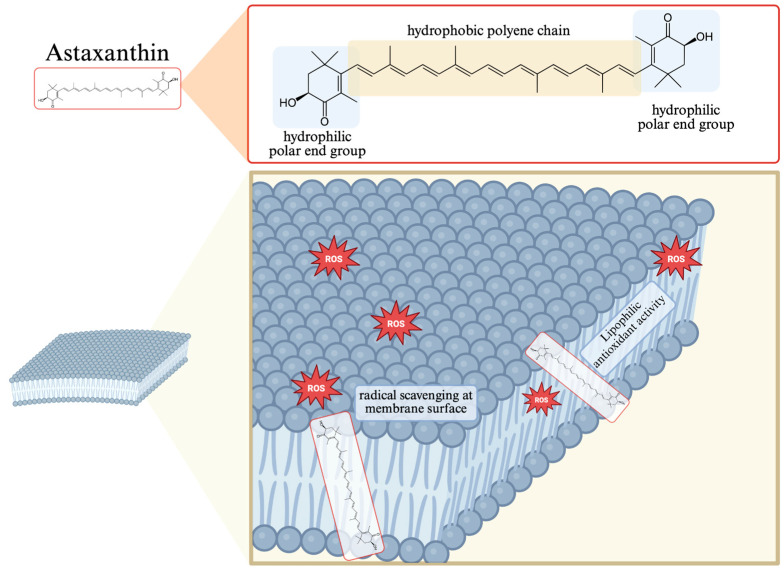
**Astaxanthin molecular structure and transmembrane orientation in a phospholipid bilayer.** Hydrophilic polar end groups scavenge reactive species at the membrane surface, whereas the hydrophobic polyene chain localizes in the lipid core, providing lipophilic antioxidant protection. Created in BioRender. Siqueira, J. (2026) https://BioRender.com/1ctkkck.

Astaxanthin (AX) is a naturally occurring xanthophyll carotenoid widely recognized for its potent antioxidant and anti-inflammatory properties. It is primarily synthesized by microalgae such as *Haematococcus pluvialis (H. pluvialis)*, *Chlorella zofingiensis (C. zofingiensis)*, and certain yeast including *Xanthophyllomyces dendrorhous (X. dendrorhous)* and as well as by some marine bacteria, including *Paracoccus carotinifaciens (P. carotinifaciens)*. Among these, *H. pluvialis* is considered the richest natural source, capable of accumulating AX up to 3.8% of its dry biomass under stress conditions [1]. Marine organisms such as salmon, krill, shrimp, and lobsters accumulate AX through the food chain, making them major dietary sources for humans. Chemically, AX (3,3′-dihydroxy-β,β′-carotene-4,4′-dione) is characterized by its conjugated polyene chain and terminal ionone rings containing hydroxyl and keto groups. AX exists in several stereoisomeric forms, including (3R,3′R), (3S,3′S), and the meso form (3R,3′S), depending on the biological source. *H. pluvialis* primarily produces the (3S,3′S) form, while *X. dendrorhous* yields (3R,3′R). These stereochemical variations influence not only its optical activity but also its bioavailability and antioxidant potential [2]. This molecular structure allows AX to span lipid bilayers, conferring both lipophilic and hydrophilic antioxidant capacities, enabling it to quench singlet oxygen and scavenge free radicals efficiently (Figure 1). Compared with other carotenoids such as β-carotene or lutein, AX demonstrates superior antioxidant stability due to the presence of both hydroxyl and keto groups at each end of the molecule [3]. Importantly, the polar–nonpolar–polar configuration of astaxanthin enables a transmembrane orientation that stabilizes lipid bilayers and protects membrane-embedded proteins, particularly within mitochondria. This structural feature distinguishes astaxanthin from other carotenoids that localize only at the membrane surface and underlies its superior efficacy in regulating cellular redox balance. This molecular structure enables efficient quenching of singlet oxygen and scavenging of free radicals, thereby contributing to mitochondrial membrane protection [4]. Despite its high bioactivity, AX’s bioavailability can be limited by its lipophilic nature. After ingestion, it is incorporated into mixed micelles in the presence of dietary lipids, absorbed via enterocytes, and transported by chylomicrons through the lymphatic system [5]. Absorption efficiency is influenced by several factors, including dietary fat content, formulation matrix (e.g., esterified vs. free form), stereoisomer composition. Co-ingestion with dietary lipids and novel encapsulation technologies has been shown to enhance AX absorption and plasma concentration levels significantly [6]. To overcome the limitations of poor absorption, various delivery systems such as nanoemulsions, liposomes, solid lipid nanoparticles (SLNs), and inclusion complexes with cyclodextrins have been developed to enhance bioaccessibility and stability [5]. Despite these advancements, inter-individual variability in absorption remains high, and further research is warranted to optimize AX delivery for clinical efficacy. Beyond its antioxidant role, AX is increasingly recognized as a metabolic modulator capable of influencing energy homeostasis and muscle function. Nutraceuticals such as AX can support metabolic health and physical performance by regulating oxidative stress, inflammation, and mitochondrial function, key processes involved in obesity, insulin resistance, and exercise adaptation. Preclinical and clinical studies demonstrate that AX enhances skeletal muscle metabolism by stimulating mitochondrial biogenesis, promoting fatty acid oxidation, and reducing lipotoxicity under metabolic stress conditions [7,8,9]. AX supplementation, especially when combined with exercise, has been shown to decrease adipokine levels, improve lipid profiles, reduce body fat, and enhance endurance and muscle regeneration in both animal and human studies [10,11,12]. Although animal and in vitro studies consistently report benefits, evidence in humans is still emerging, with some studies showing improvements in cardiometabolic markers and exercise performance, but others reporting equivocal results, highlighting the need for further well-controlled trials [13,14]. Taken together, these findings suggest that AX acts not merely as a broad-spectrum antioxidant but as a precision nutraceutical with the capacity to modulate muscle metabolism, enhance exercise adaptation, and mitigate obesity-related dysfunction through mitochondrial and redox regulation.

Among the multiple biological activities of astaxanthin, its antioxidant, anti-obesity, and muscle-related effects were selected as the main focus of this review because these domains are mechanistically interconnected through oxidative stress regulation, mitochondrial bioenergetics, and energy homeostasis, and are the most consistently supported by preclinical and clinical evidence. Thus, the aim of this review is therefore to provide a comprehensive and updated overview of AX’s mechanistic actions in muscle metabolism, exercise performance, and obesity, with a special focus on emerging delivery strategies that translate its molecular potential into clinical bioefficacy.

## 2. Mechanistic Insights: Astaxanthin in Muscle Metabolism and Mitochondrial Function

Importantly, the effects of astaxanthin on mitochondrial function are preceded by its capacity to regulate cellular redox homeostasis. Astaxanthin reduces excessive reactive oxygen species (ROS) generation and lipid peroxidation while simultaneously enhancing endogenous antioxidant defenses. This upstream redox modulation represents a critical mechanistic step that preserves cellular integrity and creates a permissive environment for subsequent mitochondrial protection, biogenesis, and metabolic adaptation. In parallel, astaxanthin modulates redox-sensitive signaling pathways involved in the antioxidant response, contributing to the maintenance of redox balance under conditions of exercise, obesity, and metabolic stress.

### 2.1. Antioxidant Action in Skeletal Muscle

Skeletal muscle is the largest organ system in the human body, accounting for approximately 40–50% of total body mass, and plays a central role in locomotion, posture, and whole-body energy metabolism. It is composed of highly specialized multinucleated fibers capable of contracting through the cyclic interaction of actin and myosin filaments, a process tightly regulated by intracellular calcium dynamics and ATP availability. Beyond its mechanical function, skeletal muscle acts as a major metabolic hub, being responsible for substantial glucose uptake, lipid oxidation, and amino acid turnover. During physical activity or metabolic stress, skeletal muscle generates reactive oxygen species (ROS) as a natural byproduct of mitochondrial respiration and enzymatic reactions. While low to moderate levels of ROS serve as signaling molecules involved in muscle adaptation and regeneration, excessive oxidative stress can impair contractile function, promote protein oxidation, and contribute to muscle fatigue and atrophy. Therefore, maintaining redox homeostasis is crucial for preserving muscle integrity and performance. In this context, dietary antioxidants have attracted growing attention as potential modulators of muscle oxidative balance. Among them, AX, a xanthophyll carotenoid with potent antioxidant and anti-inflammatory properties, has emerged as a promising compound capable of protecting skeletal muscle from oxidative damage and improving metabolic efficiency. In recent years, however, scientific research has focused on studying the protective effects of AX at the muscle level in different conditions such as sarcopenia/atrophy, physical exercise, and the modulation of fat and glucose metabolism.

In particular, AX has been shown to have potential application in the prevention of muscle injury and degeneration during sarcopenia/atrophy. In fact, sarcopenia/atrophy is a progressive and generalized loss of skeletal muscle mass and strength that typically occurs with aging, leading to reduced physical performance, frailty, and increased risk of morbidity. The pathophysiology of sarcopenia/atrophy is multifactorial and involves complex interactions between oxidative stress, chronic low-grade inflammation, hormonal alterations, and metabolic dysregulation. Excessive production of ROS and impairment of antioxidant defenses promote oxidative damage to proteins, lipids, and mitochondrial DNA, contributing to muscle cell dysfunction. Moreover, mitochondrial impairment, increased protein turnover, and capillary regression further exacerbate muscle wasting and metabolic inefficiency. Previous studies have demonstrated that antioxidant administration can reduce oxidative stress and attenuate muscle atrophy, highlighting the potential therapeutic role of antioxidants in preventing or reversing sarcopenia. In this context, compounds such as AX, with potent antioxidant and anti-inflammatory activities, have emerged as promising agents for preserving skeletal muscle mass and function. The study conducted by Kawamura et al. investigated the effects of AX, administered alone or in combination with other antioxidants such as β-carotene and resveratrol, on the process of muscle atrophy in 7-week-old male ICR mice. To induce atrophy, one hind limb of the animals was immobilized by placing a cast at the knee and ankle joints for a period of three weeks. After removal of the cast, the mice were fed for two weeks with a standard diet enriched with AX, β-carotene, resveratrol or a combination of the three compounds. The results showed that animals treated with AX alone or with the antioxidant mixture presented a significant increase in soleus muscle weight compared to controls, suggesting a protective effect of these nutrients against immobilization-induced muscle loss [15]. In the study by Kawamura et al. [15], AX was administered through a standard diet enriched with the carotenoid, indicating that the compound was delivered as a food-mixed supplement, a modality that relies on concurrent lipid intake for efficient intestinal absorption. To investigate the effects of AX on muscle atrophy, an animal model of hindlimb unloading was also used. In a study conducted by Kanazashi et al. [16], adult male Wistar rats were subjected to tail suspension for seven days to eliminate mechanical loading on the hindlimb and prevent it from contacting the floor or cage walls. During the experimental period, the animals received oral administration of AX at a dose of 50 mg/kg twice daily. The results showed that AX treatment counteracted the alterations induced by unloading, maintaining unchanged microvascular parameters such as the capillary-to-fiber ratio (C/F), the number of capillaries per fiber (CAF), capillary volume, and mean capillary diameter. These data indicate that AX supplementation, while not significantly affecting muscle mass, is able to prevent capillary regression associated with inactivity. Based on these findings, the authors hypothesized that the combination of AX and intermittent mechanical stimulation could exert a synergistic effect in preventing muscle atrophy and capillary rarefaction during unloading periods. In a subsequent experiment, rats were subjected to the same suspension protocol, but with daily release of the device for one hour, allowing normal cage movement during the dark phase. The study, extended to two weeks, confirmed that the combination of intermittent unloading and AX determined an improvement in both soleus muscle atrophy and capillary density, compared to animals treated with continuous unloading [17]. In another study, dietary AX supplementation, administered both before and during the hindlimb unloading period, showed a protective effect against soleus muscle atrophy. Compared to untreated controls, animals receiving AX incorporated directly into the diet, showed greater muscle weight and increased muscle fiber cross-sectional area (FCSA), suggesting a direct role of the compound in preserving the structural and functional integrity of the muscle during inactivity [18]. Another experimental model commonly used to study muscle atrophy is hindlimb immobilization. This procedure involves blocking a joint or bone segment using a cast or other fixation method, with the aim of limiting movement and progressively inducing contracture and reduction in muscle mass [19,20]. Alternatively, the hindlimb unloading model in rodents—obtained by placing the animals suspended head-down—is used to simulate gravitational unloading and weightlessness [21]. In an in vivo study, male Wistar rats were divided into three groups and fed a standard diet (placebo) or a diet containing 0.04% or 0.2% AX for 24 days ensuring continuous intake together with dietary lipids. Starting on the fourteenth day, the animals were subjected to hindlimb immobilization using a cast in a position of maximum plantar flexion. Results showed that AX supplementation significantly attenuated the degree of muscle atrophy compared to the control group [22]. Similarly, Maezawa et al. [23] used a similar model in seven-week-old male Wistar rats, in which the ankle joint was immobilized for two weeks. AX, administered orally at a dose of 100 mg/kg per day, via feeding tube, using a medium-chain triglyceride-based preparation, starting one week before immobilization and throughout the treatment period, resulted in a reduction in the decline in FCSA (muscle fiber cross-sectional area), indicating a protective effect against immobilization-induced atrophy [23].

Although human research on the effects of AX on muscle function is still limited, one significant study has provided interesting results. In a randomized, double-blind, placebo-controlled clinical trial, Liu et al. [24] evaluated a nutraceutical combination containing AX (12 mg), tocotrienol (10 mg), and zinc (6 mg) administered as capsules during the exercise training period, with the aim of improving strength, endurance, and motor skills in older adults. A total of 42 participants, aged 65 to 85 years, were assigned to receive the experimental formulation or a placebo for a period of four months, during which they followed a progressive treadmill training program on an incline for three months (three weekly sessions lasting 40–60 min each). Muscle strength was assessed by measuring maximum voluntary contraction (MVC) during ankle dorsiflexion, while tibialis anterior muscle mass was determined by cross-sectional area (CSA) using magnetic resonance imaging. Results showed that both groups improved 6 min walking endurance after the training period; however, subjects taking the AX-containing formulation showed significantly higher MVC and CSA values than the placebo group, suggesting a positive effect of the supplementation on strength and muscle hypertrophy. Overall, the evidence derived from in vivo studies suggested a beneficial effect of AX in preventing muscle degeneration.

Although current evidence highlights the protective effects of AX on skeletal muscle, several questions remain open. Future studies should clarify how AX influences mitochondrial biogenesis, particularly its regulation of PGC-1α and other transcriptional pathways involved in mitochondrial remodeling. Additional human trials with standardized dosing and bioavailability assessments are needed to confirm whether AX improves mitochondrial function, capillarity, or muscle mass in aging or disease conditions. It will also be important to determine whether AX acts synergistically with mechanical loading or exercise, and to define optimal intake strategies, including dose–response relationships and lipid-based delivery systems. Finally, research should investigate the effects of AX on fiber-type-specific mitochondrial adaptations, satellite cell activity, and mitophagy, to better understand its role in preserving muscle metabolic health.

### 2.2. Modulation of Fat and Glucose Metabolism in Skeletal Muscle

Skeletal muscle is a key organ for maintaining energy homeostasis, motor skills, and overall health. Under high-fat diets (HFD), this tissue is particularly vulnerable to metabolic dysfunction [25]. Excessive lipid deposition within muscle fibers leads to reduced insulin sensitivity, impaired glucose uptake, and the activation of local inflammatory processes. These events significantly contribute to the development of metabolic diseases such as obesity and type 2 diabetes mellitus [26,27]. Furthermore, the disorder in lipid metabolism is not limited to biochemical aspects, but is also associated with structural and functional changes in muscle cells, compromising their contractile efficiency and metabolic adaptation capacity [28]. Mitochondria are essential for energy production and maintaining muscle metabolism. In skeletal muscle, an HFD alters their function, reducing biogenesis, increasing fragmentation, and compromising oxidative phosphorylation [25,29]. Excess lipids also promote the formation of ROS, which damage mitochondrial structures and reduce energy capacity, contributing to the loss of muscle mass and function [26,30,31]. Improving mitochondrial biogenesis and homeostasis therefore represents a promising strategy to counteract oxidative damage and preserve skeletal muscle health.

A recent study examined the effects of an HFD on mitochondrial function and skeletal muscle, evaluating the potential protective role of astaxanthin under conditions of metabolic stress. In HFD-fed mouse models and in palmitic acid-treated C2C12 cells, astaxanthin administration did not alter body weight or plasma lipid levels, but significantly improved muscle structure and function. Astaxanthin reduced inflammation and oxidative stress, promoted mitochondrial biogenesis, attenuated mitochondrial and lipid damage, and increased antioxidant enzyme activity and ATP production. Furthermore, it limited lipid stress-induced mitochondrial fission, indicating an overall protective effect on muscle metabolism [32]. Liu et al. analyzed the impact of astaxanthin supplementation on lipid metabolism in mouse skeletal muscle, with a focus on peroxisome-proliferator-activated receptor-gamma coactivator 1-alpha (PGC-1α). Mice were divided into four groups: sedentary, sedentary treated with astaxanthin, exercise-treated, and exercise-treated with astaxanthin. After two weeks of treatment, the trained groups performed a 30 min treadmill run at moderate speed. Immediately after the exercise, intermuscular pH, plasma fatty acid levels, and muscle proteins, including PGC-1α and its targets, were assessed. The results showed that astaxanthin significantly reduced post-exercise plasma fatty acid levels and attenuated the exercise-induced decrease in muscle pH. Furthermore, treated mice had increased levels of PGC-1α and downstream regulatory proteins, indicating that astaxanthin can stimulate mitochondrial function and promote lipid oxidation, improving the efficiency of aerobic metabolism in skeletal muscle [33]. Aoi et al. (2008) demonstrated that astaxanthin supplementation improved lipid utilization during exercise in mice, prolonging running time to exhaustion [34]. Treatment increased the colocalization of fatty acid translocase with carnitine palmitoyltransferase I (CPT I) in skeletal muscle and prevented exercise-induced oxidative modifications of this protein. Furthermore, astaxanthin accelerated the reduction in body fat during exercise, suggesting a role in enhancing lipid metabolism and muscular endurance [34]. In both studies by Aoi et al. [34] and Liu et al. [33], AX was incorporated into the chow at 0.02% (*w*/*w*), a diet-mixed modality that guarantees co-ingestion with lipids. Oliva et al. (2025) demonstrated that a high-sucrose diet induced dyslipidemia, intramuscular lipid accumulation, and oxidative stress in skeletal muscle of rats [8]. Astaxanthin treatment attenuated these alterations by reducing lipogenesis, increasing CPT-1 activity and PPARα expression, and improving redox balance through the modulation of NrF2 and pNFκB p65. These results suggest a protective role for astaxanthin in preserving muscle function in conditions of metabolic syndrome [8].

Recent evidence indicates that the rise in obesity and associated diseases, including type 2 diabetes, cardiovascular disease, and some cancers, poses a serious threat to public health, significantly affecting skeletal muscle [35]. Previous studies have identified insulin resistance in skeletal muscle as a key feature of type 2 diabetes. Several transcription factors, including PPARα, PPARδ, and PPARγ, play a crucial role in regulating insulin sensitivity, glucose tolerance, lipid metabolism, and energy expenditure in skeletal muscle [36,37]. The activation of these transcription factors has been correlated with increased mitochondrial biogenesis, significantly improving muscle endurance capacity and attenuating insulin resistance in obesity and type 2 diabetes [38]. Furthermore, it is well documented that mitochondrial dysfunction and oxidative stress, often induced by excess energy and increased circulating free fatty acids, contribute to the development of insulin resistance in skeletal muscle [39]. Therefore, interventions aimed at stimulating mitochondrial biogenesis are strategic for preserving mitochondrial function and improving both insulin sensitivity and muscle energy metabolism.

In this context, astaxanthin emerges as a potent antioxidant, thanks to the presence of numerous conjugated double bonds, superior to other carotenoids. Studies such as those by Nishida et al. have shown that astaxanthin can significantly increase insulin sensitivity by promoting mitochondrial biogenesis, through the activation of the AMPK (AMP-activated protein kinase) pathway in skeletal muscle [7]. Furthermore, Huang et al. demonstrated that insulin stimulates the PI3K/Akt signaling pathway by binding to receptors on skeletal muscle cells, thereby promoting the translocation of glucose transporter type 4 (GLUT4) from intracellular compartments to the plasma membrane and which results in increased glucose uptake and metabolism [40]. Similarly, Arunkumar et al. showed that AST increases the ratio of phosphorylated Akt to Akt and promotes the translocation of GLUT4 into skeletal muscle of HFD-fed mice, activating PI3K/Akt signaling and counteracting insulin resistance [41]. Furthermore, AMPK plays a key role in promoting glucose uptake and fatty acid oxidation in skeletal muscle.

Future studies should deepen our understanding of how astaxanthin modulates the network of regulators involved in mitochondrial biogenesis, including AMPK, PGC-1α, and downstream transcription factors. In particular, it would be valuable to clarify whether AX acts primarily as an antioxidant or whether it exerts direct signaling effects on mitochondrial dynamics, fusion–fission balance, and mitophagy. Another important direction is the characterization of dose–response relationships, pharmacokinetics, and tissue-specific bioavailability of AX, especially in skeletal muscle under metabolic stress. Investigating the interaction between AX supplementation and lifestyle interventions—such as exercise or different types of diets—may also help to identify synergistic strategies to enhance mitochondrial function. Finally, translating preclinical findings into well-controlled human studies is essential to determine whether AX can effectively improve muscle metabolism, insulin sensitivity, and lipid handling in individuals with obesity, metabolic syndrome, or type 2 diabetes.

### 2.3. Effects of Astaxanthin on Mitochondrial Biogenesis and AMPK Activation

Mitochondrial biogenesis is a crucial process for maintaining skeletal muscle metabolic function, as it determines the number and efficiency of mitochondria in muscle fibers. Increased mitochondrial biogenesis improves muscle oxidative capacity, promoting ATP production through oxidative phosphorylation and optimizing the use of glucose and fatty acids as fuel. This adaptation is crucial during prolonged or high-intensity exercise, as it supports muscular endurance, reduces the accumulation of toxic metabolites, and contributes to the regulation of energy homeostasis. Key factors such as PGC-1α, AMPK, and SIRT1 coordinate this process, integrating nutritional signals and exercise to modulate mitochondrial function and muscle metabolism. Wang et al. demonstrate that AX can promote antioxidant capacity and mitochondrial biogenesis as well as reduce oxidative stress during intense HIIT (High Intensity Interval Training) exercise in mice [42]. Recent studies have highlighted the protective role of AX on skeletal muscle subjected to metabolic stress induced by high-fat diets (HFD) or accumulation of fatty acids such as palmitate. The results show that AX significantly reduces muscle damage and improves muscle tissue function, while it does not affect body weight or serum lipid levels. The authors observed that AX suppresses inflammatory gene expression, stimulates proteins involved in mitochondrial biogenesis, and limits mitochondrial damage in in vivo and in vitro models. Furthermore, AX inhibits mitochondrial fragmentation in C2C12 cells treated with palmitate. The authors thus confirm that AX supports skeletal muscle structure and function by promoting mitochondrial biogenesis and modulating oxidative stress and inflammation, indicating its therapeutic potential in conditions of metabolic stress [32]. Chen et al. have shown that AX stimulates the expression of proteins involved in both muscle regeneration and mitochondrial biogenesis, suggesting an enhancement of muscle oxidative and metabolic capacity. Specifically, AX upregulated peroxisome proliferator-activated receptor-γ coactivator 1-alpha (PGC-1α) and mitochondrial transcription factor A (TFAM), key regulators of mitochondrial DNA replication and transcription, as well as oxidative phosphorylation (OXPHOS) complex proteins, resulting in increased mitochondrial DNA content and ATP production. In parallel, AX promoted the expression of myogenic regulatory factors, including MyoD and myogenin, which are critical for satellite cell activation and myofiber regeneration.

Together, these findings indicate that AX enhances skeletal muscle recovery and function through PGC-1α/TFAM-mediated mitochondrial biogenesis and activation of muscle-specific differentiation pathways [12]. Furthermore, in vitro studies on C2C12 cells confirm that AX promotes myoblast differentiation and the formation of new mitochondria, underscoring the key role of mitochondrial biogenesis as a mechanism through which AX improves skeletal muscle function and recovery in obese conditions [12]. Furthermore, scientific literature highlights how AX, compared to other exogenous antioxidants, has a greater capacity to reduce ROS and preserve the structural integrity of mitochondria in cyclists trained for aerobic endurance performance when AX was administered encapsulated at 4 mg/day for 28 days [43,44]. In particular, it appears to promote mitochondrial biogenesis, by enhancing the oxidative capacity of muscle fibers and improving energy efficiency during prolonged efforts. These effects translate into improvements in aerobic performance, regulation of submaximal heart rate, post-exercise recovery, and increased endogenous antioxidant capacity, suggesting that AX may act as an adjuvant to endurance training by stimulating key mitochondrial adaptations. Recent evidence suggests that AX can significantly improve skeletal muscle metabolism in conditions of high-fat diet-induced insulin resistance. Specifically, the landmark study demonstrated that mice treated with AX exhibited significant activation of AMPK, a kinase sensitive to cellular energy levels that coordinates metabolism and promotes mitochondrial energy production. AMPK activation by AX promotes mitochondrial biogenesis, increasing oxidative capacity and muscle fatty acid metabolism. The role of AMPK was confirmed in vitro using C2C12 cells genetically depleted of AMPK, where AX’s stimulatory effect on mitochondrial biogenesis was completely lost [7]. These results highlight how AX can modulate insulin sensitivity and skeletal muscle metabolic function through stimulation of AMPK-mediated mitochondrial biogenesis. Future studies should aim to clarify several open questions regarding the mechanisms and applications of AX in regulating skeletal muscle mitochondrial biogenesis. First, it will be important to define the dose–response relationship and optimal formulations (e.g., encapsulated vs. free AX) required to maximize mitochondrial adaptations in humans, since current evidence is limited and heterogeneous. Moreover, the cellular signaling pathways upstream and downstream of AMPK, PGC-1α, and SIRT1 remain only partially understood; dissecting how AX integrates into these networks—especially in the context of metabolic stress—will help explain its tissue-specific effects. Another promising avenue is the investigation of AX’s role in mitochondrial dynamics, including fusion, fission, and mitophagy, to determine whether its protective effects extend beyond the stimulation of biogenesis to broader control of mitochondrial quality. Additionally, research should explore whether AX can modulate satellite cell function and muscle regeneration in different physiological and pathological contexts, such as aging, sarcopenia, or metabolic syndrome. Longitudinal human studies are also needed to verify whether AX supplementation can translate into measurable improvements in muscle performance, insulin sensitivity, and metabolic flexibility, and whether these effects synergize with various training modalities (HIIT, endurance, resistance training). Finally, investigating the interaction between AX and dietary patterns—particularly high-fat diets or low-energy availability—could reveal how nutritional context influences AX’s efficacy on mitochondrial health.

### 2.4. Protective Role of Astaxanthin on Mitochondria in Skeletal Muscle

**Figure 2:**
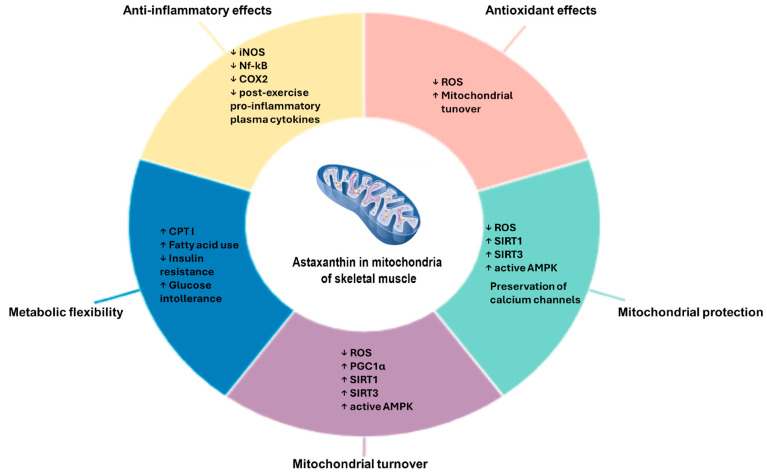
**Circular diagram of the main effects of astaxanthin on mitochondria in skeletal muscle.** Arrows pointing upward (↑) indicate an increase, while arrows pointing downward (↓) indicate a reduction.

Physiologically, AX has a protective role in the muscle of fish, particularly in salmon, where it protects muscle membranes during extremely demanding physical exertion, such as during marine migrations [4]. Based on this role, much of the research in the literature has focused on elucidating the mechanisms underlying this function and investigating the potential protective role of this carotenoid when used as a nutraceutical during and after physical exercise in humans. Numerous pieces of evidence have linked the use of AX in humans to a reduction in key markers of oxidative stress and inflammation [4,45]. AX has been shown to buffer mitochondrial overload, which can be induced by physical exercise, as exercise inherently promotes ROS formation and mobilizes fuels within the mitochondria, but also by an HFD, as observed in obesity, which continuously stimulates the mitochondrial network in ATP production, fatty acid synthesis, and the consequent generation of ROS (Figure 2) [4,46]. Under these conditions, mitochondrial dynamics are also accelerated to balance the demand for new mitochondria with the removal of those that are functionally exhausted.

Early studies on the mitochondrial protective properties of AX primarily focused on its ability to safeguard membranes, preventing lipid peroxidation and, consequently, ferroptosis. Indeed, it has been demonstrated that AX can protect mitochondrial respiratory chain activity against Fe^2+^-induced lipid peroxidation, even in mitochondria isolated from vitamin E-deficient rats, thereby counteracting the effects of vitamin E deficiency [47]. Indeed, AX possesses a hydrophobic conjugated polyene backbone with terminal polar groups, which enables it to effectively quench free radicals both within and outside the mitochondrial membrane. Moreover, AX exhibits a strong affinity for the superoxide anion as well as for peroxyl radical intermediates. By quenching superoxide and converting it back to ground-state oxygen, while simultaneously scavenging and deactivating peroxyl radical intermediates, AX effectively mitigates oxidative damage within the mitochondria [48,49].

Another protective aspect of AX in mitochondria relates to its ability to preserve the structure of calcium channels while maintaining the integrity of endoplasmic reticulum membranes (Figure 2). This is particularly important because mitochondria are susceptible to disturbances of Ca^2+^ homeostasis. Following excessive ROS exposure and endoplasmic reticulum disruption, mitochondrial membranes become increasingly permeable to Ca^2+^, resulting in impaired mitochondrial function and decreased oxidative phosphorylation efficiency. Over time, mitochondria may release pro-apoptotic factors to initiate apoptosis [48,50].

On the other hand, AX can enhance mitochondrial structure and performance by modulating the turnover of these organelles, primarily promoting mitochondrial biogenesis in myotubes. This process is closely linked to oxidative metabolism and to a type of slow, sustained effort that muscle fibers are particularly adapted for. Indeed, fibers with a higher number of myotubes—namely, type I oxidative fibers—are capable of slow, sustained contractions and are more resistant to external stressors and physical exercise [51]. In the context of mitochondrial protection, the pro-biogenic role of AX is also protective, as it distributes oxidative effort across a larger mitochondrial network, thereby preventing the accumulation of excess fuel and ROS. In particular, as mentioned before, it can stimulate the expression of PGC-1α, the master regulator of mitochondrial biogenesis [42]. The increase in PGC-1α expression in muscle induced by AX is dependent on AMPK, as demonstrated in C2C12 myotubes, where AMPK silencing completely abolishes the AX-induced activation of PGC-1α [7]. Moreover, in the skeletal muscles of AX-treated obese and lean mice, the expression levels of Sirtuins, such as Sirt1 and Sirt3, were upregulated (Figure 2). Nampt expression was also increased, consistent with its known association with AMPK activation [7]. Furthermore, intracellular NAD^+^ levels were significantly elevated in C2C12 myoblasts treated with AX compared with the vehicle control group. These findings suggest that AX enhances mitochondrial biogenesis and energy metabolism through an AMPK-Sirtuin-PGC-1α signaling axis, likely involving the de novo synthesis of NAD^+^ [4]. In mice fed an HFD, AX has been shown to promote mitochondrial remodeling, leading to enhanced oxidative capacity and fatty acid metabolism. This is accompanied by an increase in mitochondrial mass, resulting from AMPK-mediated mitochondrial biogenesis, allowing mitochondria to better manage the surplus of available energy sources. Moreover, AX improves insulin resistance and glucose intolerance, effects that are also associated with AMPK activation [7].

This ability of AX also contributes to promoting muscle regeneration in cases of muscle injury [32]. Such effects can occur under physiological conditions, such as physical exercise, but also under pathological conditions, such as obesity-induced muscle damage. In this regard, it has been shown that in C2C12 myotubes, AX promotes in vitro differentiation as well as regeneration in CTX-induced injury models of HFD-fed obese mice [12]. Overall, muscle regeneration following injury is promoted by AX through multiple mechanisms, including the modulation of cellular differentiation and the enhancement of mitochondrial biogenesis, which together contribute to improved cell survival.

This multifaceted mitochondrial protective function, encompassing enhanced mitochondrial biogenesis and oxidative phosphorylation, as well as improved muscle response to injury, positions AX as a nutraceutical capable of promoting metabolic adaptation in muscle, thereby increasing metabolic flexibility, particularly in response to exercise. Indeed, AX supplementation was found to upregulate CPT I expression and increase the colocalization of fatty acid translocase in skeletal muscle, thereby enhancing fatty acid utilization during exercise (Figure 2). At the same time, AX downregulates lipogenic genes, including SREBP-1c [8]. By shifting substrate preference toward lipid metabolism, AX supports sustained energy production during endurance activities, helps delay fatigue, and may improve overall exercise performance. This enhanced reliance on fat over carbohydrates could also offer therapeutic potential for individuals with metabolic disorders, such as obesity or type 2 diabetes, where optimizing lipid metabolism is crucial [52,53]. Conversely, there is evidence that AX enhances glucose uptake, promoting its utilization under conditions of insulin resistance and diabetes. Although the clinical profile of AX is still being actively investigated, it appears to play a critical metabolic role by regulating both glucose and fatty acid utilization, thereby contributing to improved metabolic balance [54]. Evidence in the literature highlights that the effects of AX vary depending on the type of stressors and stimuli imposed on the muscle. In this context, during physical exercise, AX promotes fatty acid mobilization, a feature particularly appealing in clinical settings where the goal of exercise is weight reduction.

### 2.5. Astaxanthin as a Modulator of Inflammatory Responses

In various physiological and pathological contexts, AX has demonstrated anti-inflammatory effects by modulating the molecular regulators of inflammation, including COX-2, iNOS, and NF-κB (Figure 2). Interestingly, some evidence also points to microglial contexts, where AX has been shown to suppress the production of inflammatory mediators in LPS-stimulated BV-2 microglial cells, suggesting potential neuroprotective effects [55]. Within the scope of this review, AX has also been reported to suppress inflammation secondary to excessive physical activity [56]. In a study by Nieman et al., short-term AX supplementation (8 mg/day for 4 weeks) was shown to enhance the recovery of twenty plasma immunoglobulins within 24 h following prolonged exercise, such as a 2.25 h running bout. Athletes receiving AX exhibited faster normalization of post-exercise pro-inflammatory plasma cytokines compared to the placebo group. These findings provide a more comprehensive view of AX’s beneficial effects, positioning this carotenoid as a potential supplement to support both muscular and systemic recovery [57]. Furthermore, the anti-inflammatory effects of AX have also been demonstrated following high-intensity, short-duration exercise. Four weeks of supplementation with this nutraceutical reduced serum levels of IL-1β, cortisol, and uric acid [58].

AX exerts anti-inflammatory effects through multiple molecular mechanisms. A central pathway involves the mitigation of oxidative stress, which plays a pivotal role in modulating inflammatory responses. Additionally, AX has been shown to inhibit the NF-κB signaling pathway, thereby reducing the expression of downstream pro-inflammatory mediators such as COX-2 and iNOS [59]. These combined mechanisms highlight AX’s multifaceted capacity to attenuate inflammation, both systemically and at the tissue level, without eliciting adverse effects, making it a promising nutraceutical for supporting immune and inflammatory homeostasis.

For the sake of completeness, it should be noted that the effects of AX supplementation on inflammation reported in the literature are not entirely consistent. In some studies, no significant improvements in inflammatory markers were observed following AX treatment under exercise conditions. Nevertheless, overall muscle performance appears to be enhanced with AX supplementation, even with short-term use, although this does not always correspond to measurable changes in inflammatory biomarkers. Importantly, supplementation has never been associated with adverse effects and is generally well tolerated [60].

Overall, the evidence discussed highlights several promising avenues for future research. First, further studies are needed to clarify the molecular mechanisms through which astaxanthin modulates oxidative stress and inflammatory signaling, particularly the interplay between ROS handling, AMPK–Sirtuin–PGC-1α activation, and downstream inflammatory pathways. It will also be important to explore how AX-induced changes in mitochondrial biogenesis and turnover influence immunometabolic processes within muscle, including cytokine production and the resolution of inflammation. Another key direction is to understand how different physiological and pathological stressors, such as endurance exercise, high-fat feeding, or obesity-related muscle damage, shape the anti-inflammatory and mitoprotective actions of AX. These insights could help identify the contexts in which AX is most effective. Finally, more translational work is needed to determine whether AX can provide clinical benefits in conditions characterized by metabolic dysfunction and chronic low-grade inflammation, such as obesity, type 2 diabetes, or impaired muscle regeneration. Clarifying these aspects will be essential for defining the therapeutic potential of astaxanthin as a modulator of metabolic and inflammatory responses in skeletal muscle.

## 3. Astaxanthin and Obesity: Multi-Organ Metabolic Effects

### 3.1. Effects on Adipose Tissue

It is noteworthy that the beneficial effects of AX on redox balance and mitochondrial function in skeletal muscle, discussed in the previous sections, are closely interconnected with its anti-obesogenic actions. By promoting metabolic re-equilibration in adipose and hepatic tissues, AX contributes to the maintenance of muscle homeostasis and enhances skeletal muscle adaptation during exercise and in conditions of insulin resistance. In this integrative context, the following section highlights the principal mechanisms and evidence supporting the anti-obesity effects of AX.

The excessive expansion of white adipose tissue (WAT) is a defining pathological feature of obesity, which Ng et al. (2025) identify as a major global health crisis [61]. The predominant etiological factor of obesity is a chronic positive energy balance (energy intake persistently exceeding expenditure), largely associated with Western diet patterns [62]. Chronic exposure to these diets promotes a sustained influx of triglycerides into adipocytes, leading to both hypertrophy and hyperplasia of these cells within WAT [63].

Excessive adipocyte expansion leads to local hypoxia, cellular necrosis, and recruitment of immune cells, triggering a systemic redox inflammatory imbalance, affecting other organs and tissues such as the heart, liver, kidneys, and skeletal muscle [64,65,66].

AX is a potent xanthophyl carotenoid with antioxidant capacity, reported to be up to 100-fold higher than vitamin E (alpha-tocopherol) and other carotenoids, combined with pronounced anti-inflammatory and immunomodulatory properties [67,68]. Experimental evidence from both cell culture and diet-induced obesity models provides mechanistic insight into the anti-obesogenic properties of AX. In Ldlr^−^/^−^ mice fed a high-fat, high-cholesterol diet for 16 weeks, alternate-day gavage supplementation with AX (70 mg/kg) reduced leucocyte infiltration in WAT by 34%, improved insulin sensitivity, and enhanced glucose tolerance [69]. Interestingly, AX supplementation reduced WAT mass and increased skeletal muscle mass independently of total body weight changes.

In vitro, treatment of 3T3-L1 adipocytes with AX (0–25 µg/mL, 48 h) inhibited glycerol-3-phosphate dehydrogenase (GPDH) activity and suppressed the expression of key lipogenic genes (PPARγ, FAS, ACC) and fatty acid transporters (aP2, CD36, LPL), thereby attenuating intracellular triglyceride accumulation [70]. These effects indicate a context-dependent modulation of peroxisome proliferator-activated receptor gamma (PPARγ) activity, whereby astaxanthin limits pathological adipogenesis and inflammatory signaling while preserving PPARγ-associated insulin sensitivity and adipose tissue metabolic function under obesogenic conditions.

Complementarily, Nawaz et al. (2021) demonstrated that dietary AX supplementation (0.02%) in male C57BL/6J mice fed an HFD for 24 weeks downregulated oxidative stress markers (heme oxygenase 1 gene–Hmox1), M1 macrophage infiltration, and pro-inflammatory gene expression (Ccr2, IL1-β, IL-6, Nlrp3, Nos2, and TNF-α), while upregulating anti-inflammatory mediators, as interleukine-10 (IL-10), mitochondrial sirtuin 3 (Sirt3), the antioxidant enzyme catalase, and PPARγ [71]. Furthermore, AX also preserved adipocyte stem and progenitor cells, maintained vascular integrity, and enhanced hypoxia response mediated by hypoxia-inducible factor 2α (Hif2a), thereby preventing maladaptive adipose tissue remodeling [71].

Recent evidence also identifies the gut microbiota as an additional target mediating the anti-obesity actions of AX. In HFD-fed male C57BL/6J mice, AX supplementation (25 mg/kg/day) during the final 5 weeks of a 15-week intervention significantly reduced body weight, total cholesterol, triglycerides, and LDL-cholesterol levels, while markedly upregulating uncoupling protein 1 (UCP1) expression in brown adipose tissue (BAT) and promoting browning of inguinal WAT, represented by increased UCP1 and PRDM16 levels [72]. These thermogenic adaptations were accompanied by AX-induced remodeling of the gut microbiota, characterized by restored microbial diversity and a reduced *Firmicutes/Bacteroidetes* ratio. More specifically, AX supplementation enhances the abundance of anaerobic genera such as *Alistipes* and *Alloprevotella*, both known to produce short-chain fatty acids (acetate, propionate, butyrate) and amino acid–derived metabolites (e.g., pyruvate, isoleucine) that act as signaling molecules for mitochondrial activation and UCP1 transcription in adipose tissue [73,74]. These metabolites stimulate β-oxidation and thermogenic pathways via PGC-1α and PRDM16, promoting a functional shift toward oxidative metabolism in brown and beige adipocytes [75,76].

AX-driven microbial remodeling appears strongly diet-dependent, showing greater modulation under high-fat diet conditions, where AX reverses HFD-induced dysbiosis and inflammation by enriching *Alistipes*, *Alloprevotella*, and *Akkermansia*, while suppressing opportunistic taxa linked to endotoxemia [73,75,76]. These microbial shifts coincide with elevated SCFA levels and increased expression of UCP1, CPT1B, and SIRT1 in adipose depots, indicating that AX promotes thermogenesis through a gut microbiota–metabolite–UCP1 regulatory axis that integrates intestinal and systemic energy homeostasis [73,75,76].

This emerging microbiota–metabolome–thermogenesis axis underscores the multifactorial and integrative nature of AX’s anti-obesogenic mechanisms.

Clinical evidence proposes that supplementation with AX is effective in modulating adipokine levels and risk factors associated with obesity, particularly when combined with functional and high-intensity exercise.

Saeidi et al. (2023) demonstrated that obese men supplemented with 20 mg/day of AX combined with 12 weeks of high-intensity functional training (three 60 min sessions/week) presented body weight, body mass index and fat mass reduction [10]. Regarding the glycemic and lipid profiles, the intervention led to improved levels of HDL-C, LDL-C, total cholesterol, and triglycerides. Furthermore, there was observed an improvement in glucose, insulin, and insulin resistance. In addition, the levels of the adipokines C1q/TNF-related protein 2 (CTRP2) and C1q/TNF-related protein 9 (CTRP9), and the growth factors growth differentiation factor 8 (GDF8) and growth differentiation factor 15 (GDF15) were significantly reduced [10]. The evidence suggests that AX supplementation, both alone and in combination with exercise, is able to ameliorate risk factors positively associated with dyslipidemias, type 2 diabetes, and cardiovascular impairment.

Collectively, these findings highlight the integrative role of AX in coordinating metabolic and inflammatory responses across multiple tissues, pointing to the liver as a key site for its downstream regulation of lipid metabolism.

### 3.2. Integrated Metabolic Axis

Beyond its direct actions on skeletal muscle, AX also exerts systemic regulatory effects along the liver–adipose tissue–muscle axis, with the liver acting as a central metabolic hub of energy redistribution and lipid handling across peripheral tissues [77]. In this context, AX plays a crucial role in maintaining hepatic lipid homeostasis, thereby contributing to systemic metabolic balance in obesity [78,79]. In HFD models, AX attenuates hepatic triglyceride and cholesterol accumulation by downregulating key lipogenic factors such as sterol regulatory element-binding protein 1c (SREBP-1c), acetyl-CoA carboxylase (ACC), fatty acid synthase (FAS), and stearoyl-CoA desaturase-1 (SCD-1), while upregulating β-oxidation-related genes, including carnitine palmitoyltransferase 1 (CPT1) and peroxisome proliferator-activated receptor alpha (PPARα) [73]. AX further enhances the expression of liver X receptor alpha (LXRα) and bile acid synthesis enzymes such as cholesterol 7α-hydroxylase (CYP7A1) and cytochrome P450 27A1 (CYP27A1), promoting cholesterol conversion into bile acids and preventing hepatic lipid overload. These effects are accompanied by elevated peroxisome proliferator-activated receptor gamma coactivator-1 alpha (PGC-1α) levels, suggesting improved mitochondrial fatty acid oxidation and energy turnover [80].

At the molecular level, these improvements in hepatic lipid handling are closely linked to the ability of AX to modulate fatty acid composition and protect membrane lipids from oxidative damage. AX may influence Polyunsaturated fatty acid (PUFA) metabolism through the modulation of fatty acid composition and the prevention of oxidative degradation rather than by directly stimulating PUFA biosynthesis [81]. Dietary AX supplementation significantly alters hepatic and muscular fatty acid profiles, reducing hepatic arachidonic acid (ARA) and long-chain *n*-3 PUFA such as eicosapentaenoic acid (EPA), while increasing precursor fatty acids including linoleic (18:2*n*-6) and α-linolenic acids (18:3*n*-3), without significantly affecting Docosahexaenoic acid (DHA) levels [81].These changes are accompanied by a marked separation of hepatic fatty acid profiles in response to higher AX intake, indicating a dose-dependent remodeling of lipid metabolism. Mechanistically, these effects appear to be linked to the antioxidant properties of AX, as excessive PUFA synthesis under low antioxidant conditions has been proposed to enhance lipid peroxidation and the accumulation of toxic oxidation products. By limiting lipid peroxidation, AX may reduce the need for compensatory PUFA biosynthesis and protect long-chain PUFAs from excessive oxidative decomposition [82,83]. In line with this, AX has been shown to preserve membrane integrity by preventing PUFA peroxidation in membrane phospholipids, in contrast to nonpolar carotenoids that disrupt bilayer organization and exacerbate lipid oxidation. This membrane-protective role extends to the inhibition of ferroptosis, an iron-dependent form of PUFA-driven lipid peroxidation, thereby contributing to the protection of cellular and organ function across tissues in metabolic, cardiovascular, and neurodegenerative contexts [84,85].

Building on its membrane-protective and lipid-modulating effects, AX also shifts the PPARα/PPARγ balance toward a catabolic profile over lipid storage, thereby promoting fatty acid oxidation and limiting ectopic lipid accumulation [78]. By reducing hepatic macrophage infiltration and inhibiting JNK/p38 MAPK and NF-κB signaling, AX mitigates inflammatory responses and improves insulin sensitivity in hepatocytes. Through these anti-inflammatory effects, AX helps restore the phosphorylation of Akt and insulin receptor substrate-1 (IRS-1) in insulin-sensitive tissues, thereby improving systemic insulin signaling. Activation of the AMPK–SIRT1–PGC1α axis further enhances fatty acid oxidation, mitochondrial biogenesis, and glucose utilization, contributing to the restoration of metabolic flexibility by the ability of tissues to shift between lipid and carbohydrate oxidation according to energy demand [86]. Restoration of such substrate adaptability is critical to preventing metabolic inflexibility, a hallmark of obesity and type 2 diabetes.

Mitochondrial homeostasis has also emerged as a key target of AX in the liver. Activation of the AMPK-Sirtuins-PGC1 pathway promotes mitochondrial biogenesis and function while regulating fission, fusion, and mitophagy processes, ultimately reserving organelle quality [86]. These hepatic mitochondrial adaptations not only enhance lipid oxidation locally but also contribute to the systemic coordination of energy metabolism along the liver-adipose tissue–muscle axis.

Clinical findings support these molecular mechanisms: in individuals with prediabetes and dyslipidemia, AX supplementation reduced total and LDL cholesterol levels and cardiovascular risk markers, including fibrinogen, L-selectin, and fetuin-A [79].

**Figure 3:**
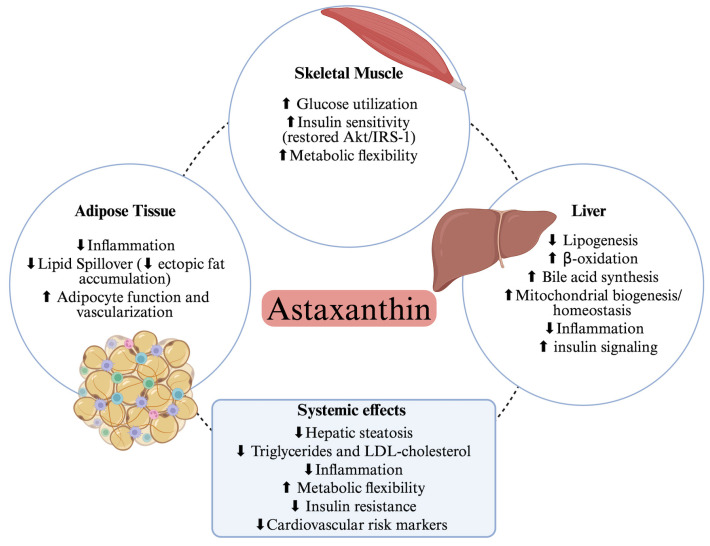
**Schematic representation of the main actions of astaxanthin on metabolic organs and systemic regulation.** Arrows pointing upward (↑) indicate an increase, while arrows pointing downward (↓) indicate a reduction. Created in BioRender. Siqueira, J. (2025) https://BioRender.com/w33jcxh.

Collectively, these hepatic actions prevent steatosis and limit lipid spillover to adipose and muscle tissues, alleviating insulin resistance. These integrated effects along the liver–adipose–muscle axis are summarized in Figure 3. By restoring hepatic lipid handling, bile acid metabolism, and mitochondrial oxidative capacity, AX reinforces the functional integration of the liver–adipose–muscle axis, a key determinant of metabolic homeostasis disrupted in obesity.

Beyond peripheral metabolic tissues, emerging evidence suggests that AX may also exert beneficial effects at the brain level in obesity-related contexts. Although clinical studies specifically addressing AX effects on the brain in obesity are still lacking, preclinical data indicate that AX is able to cross the blood–brain barrier and accumulate in metabolically relevant brain regions, including the hippocampus [87]. In models of HFD feeding, diabetes, and insulin resistance, AX supplementation attenuates neuroinflammation, oxidative stress, and cognitive impairment, while modulating nutrient-sensing, inflammatory, and neuronal survival pathways [88,89,90,91]. These neuroprotective effects appear to be mechanistically linked to AX-mediated suppression of NF-κB signaling and preservation of mitochondrial function, whereas improvements in Akt-dependent insulin signaling are primarily observed in peripheral tissues, suggesting that central actions of AX may complement its peripheral metabolic effects along the liver–adipose–muscle axis. Notably, in models combining metabolic dysfunction and neurodegeneration, AX reversed diabetes-induced cognitive deficits while concomitantly improving hepatic insulin signaling and modulating brain nutrient-sensing and inflammatory pathways, highlighting the tight interplay between peripheral and central metabolic regulation [88].

## 4. Astaxanthin and Exercise Adaptation

Evidence from preclinical and clinical studies indicates that AX supplementation exerts meaningful effects on muscle metabolism, exercise adaptation, oxidative stress, and recovery, although the magnitude and consistency of these effects depend on dose, duration, fitness level, and metabolic status.

During strenuous exercise, excessive reactive oxygen and nitrogen species (RONS) production can impair muscle function and lead to oxidative stress, inflammation, and fatigue. AX’s unique ability to integrate into cellular and mitochondrial membranes enables it to protect phospholipid structures from peroxidation, thereby preserving energy metabolism and redox balance during exercise challenges [92]. Animal studies consistently show that AX supplementation improves endurance capacity and delays fatigue (Table 1). In mice, AX increased fat utilization and running time to exhaustion by enhancing mitochondrial β-oxidation and preventing oxidative modification of carnitine palmitoyltransferase I (CPT-I), a key enzyme in lipid metabolism [34]. Supplementation also delayed physical exhaustion in Wistar rats by 29% and improved antioxidant defenses, including mitochondrial superoxide dismutase and glutathione peroxidase activity in skeletal muscle [93]. Moreover, AX effectively mitigates oxidative damage and inflammation associated with exhaustive exercise. In mice, dietary AX attenuated exercise-induced skeletal and cardiac muscle injury by reducing 4-hydroxy-2-nonenal (4-HNE) and 8-hydroxy-2′-deoxyguanosine (8-OHdG) accumulation and limiting neutrophil infiltration [94]. Notably, dose–response effects appear non-linear: very high doses may downregulate endogenous antioxidant enzymes, as observed in swimming-trained mice [95], suggesting an optimal dosing range rather than a simple dose-dependent improvement.

Human trials provide partially convergent evidence regarding the impact of AX on performance, metabolic adaptation, muscle damage, and recovery, as summarized in Table 2. Findings consistently highlight that the effects of AX vary according to training status, supplementation dose, duration, and the physiological demands of the exercise model. In humans, findings on performance and metabolic adaptation differ according to training status. In recreational or untrained individuals, several trials report improvements in exercise capacity, lipid oxidation, and endurance. Short-term supplementation increased fat oxidation and improved 40 km time-trial performance in recreational cyclists [44], while acute high-dose supplementation enhanced time-to-exhaustion and reduced CK and MDA in young adults [60]. In older adults, AX combined with aerobic exercise increased fat oxidation and endurance capacity [52], and combined with strength training increased muscle strength and cross-sectional area [24]. In contrast, highly trained athletes such as competitive cyclists did not experience improvements in fat oxidation or time-trial performance with low-dose (20 mg/day, 4 weeks) supplementation [96]. These findings suggest that AX may preferentially benefit populations with lower baseline mitochondrial efficiency or greater oxidative burden.

Results concerning muscle damage and recovery are heterogeneous. In competitive cyclists, AX did not reduce exercise-induced cTnT release or oxidative stress biomarkers [97]. Similarly, resistance-trained athletes showed no significant improvements in CK, DOMS, IL-6, or inflammatory markers following eccentric exercise [98]. However, longer-term supplementation in elite soccer players reduced post-exercise CK and AST levels, reflecting decreased muscle damage and improved redox status [99]. In addition, a randomized trial found that 12 mg/day AX for four weeks significantly lowered subjective DOMS in resistance-trained men, although without corresponding changes in objective performance metrics [100]. A recent proteomic study further demonstrated that four weeks of AX (8 mg/day) helped restore post-exercise concentrations of immune-related plasma proteins and immunoglobulins after strenuous endurance exercise, indicating improved immune homeostasis and recovery [57].

While animal evidence consistently demonstrates improved endurance, lipid utilization, and mitochondrial protection, human studies have produced mixed results. In young athletes, AX may reduce post-exercise lactate and muscle damage markers, but effects on maximal oxygen uptake, anaerobic threshold, and performance are inconsistent [101]. Comparatively, the effect size is generally smaller in humans than in animal models, likely due to differences in baseline antioxidant capacity and training-induced adaptations [102].

Nevertheless, reviews and mechanistic evidence converge on AX’s role in enhancing mitochondrial efficiency, attenuating exercise-induced oxidative damage, and modulating redox-sensitive pathways that support endurance adaptations [13,48,97]. Recent human studies also corroborate potential metabolic benefits. In obese men, AX combined with high-intensity functional training improved VO_2_max, reduced adipokines (CTRP2, CTRP9, GDF8), and enhanced metabolic flexibility [10]. In resistance-trained males, 12 mg/day for six weeks reduced CK, LDH, and DOMS, accelerating recovery [100]. Parallel evidence from preclinical models strengthens these observations. In a mouse HIIT (High Intensity Interval Training) model, AX supplementation upregulated PGC-1α, NRF1, and TFAM, promoting mitochondrial biogenesis and oxidative remodeling [42]. In obesity-induced metabolic dysfunction, AX enhanced AMPK activation, ATP production, and mitochondrial resilience [32], further supporting its potential role in improving muscle oxidative capacity and metabolic health.

Older adults appear particularly responsive to AX supplementation. In elderly adults, 12 mg/day AX combined with functional or high-intensity interval training significantly increased tibialis anterior muscle strength and endurance compared to placebo, indicating improved muscle adaptation with concurrent exercise stimuli [24]. Moreover, AX enhances metabolic flexibility and oxidative efficiency when combined with endurance training [52]. Given that aging is associated with mitochondrial dysfunction and heightened oxidative stress, these effects may reflect a restoration of impaired adaptive signaling pathways rather than a purely ergogenic outcome.

Recent meta-analyses strengthen and contextualize these findings. A quantitative synthesis of 11 RCTs reported significant improvements in aerobic performance and fat oxidation with medium-to-high doses administered over longer durations [103]. A second meta-analysis focusing on athletes found increases in total antioxidant capacity and potential enhancements in cycling performance [104]. Collectively, these syntheses support the view that AX exerts consistent effects on redox modulation and oxidative metabolism, with more variable outcomes on physical performance.

**Table 1:** Impact of astaxanthin supplementation on exercise-induced adaptations in animal models.

Study (Year)	Model/Subjects	Dose/Duration	AX Formulation and Administration	Main Outcomes	Key Findings	References
Aoi et al., 2008	Mice	Astaxanthin in diet (~3% extract), 4 weeks	Dietary supplementation; AX mixed into standard rodent chow	β-oxidation, CPT-I oxidation	↑ fat use, ↑ endurance, ↓ CPT-I oxidation	[34]
Polotow et al., 2014	Wistar rats	1 mg/kg/day, 45 days	Oral gavage, AstaReal^®^ biomass stock solution prepared in mineral oil	SOD, GPx, redox balance	↑ SOD/GPx, ↓ oxidative stress, ↑ exhaustion time	[93]
Aoi et al., 2003	Mice	20 mg/kg/day, 3 weeks	AX mixed into powdered chow (CE-2) (oral via feed)	4-HNE, 8-OHdG, inflammation	↓ oxidative injury, ↓ neutrophil infiltration	[94]
Wang et al., 2023	Mice (HIIT)	10 mg/kg/day, 2 h prior to exercise for 6 weeks	Oral gavage; AX dissolved in olive oil	PGC-1α, NRF1, TFAM, oxidative stress marker	↑ mitochondrial biogenesis, ↓ exercise-induced oxidative stress, ↑ antioxidant capacity	[42]
Nishida et al., 2020	Obese mice	0.02% diet, 8 weeks	Dietary supplementation; commercially available astaxanthin powder pre-mixed into normal chow or high-fat diet.	AMPK, PGC-1α	↑ AMPK activation, ↑ oxidative capacity, improved metabolic and redox homeostasis	[7]
Li et al., 2025	Obese mice	0.02% diet, 12 weeks	Oral gavage; AX dissolved in hydroxypropyl-β-cyclodextrin	AMPK, ATP	↑ AMPK, ↑ ATP, ↑ mitochondrial resilience	[32]
Zhou et al., 2019	Swimming mice	5–30 mg/kg/day, 4 weeks, 2 h prior to swimming training	Oral gavage; AX dissolved in olive oil	Nrf2, antioxidant enzymes	High doses ↓ antioxidant enzymes	[95]

Arrows pointing upward (↑) indicate an increase, while arrows pointing downward (↓) indicate a reduction in the reported biological outcomes.

**Table 2:** Impact of astaxanthin supplementation on human exercise adaptation.

Study (Year)	Model/Subjects	Dose/Duration	AX Formulation and Administration	Main Outcomes	Key Findings	References
Performance and Metabolic Adaptation						
Res et al., 2013	Trained cyclists	20 mg/day, 4 weeks	Oral gelatin capsules; *Haematococcus pluvialis* extract dissolved in sunflower oil, with added vitamin C (60 mg/capsule) and vitamin E (10 mg/capsule); ingested with breakfast and dinner.	Fat oxidation, TT performance	No ergogenic effect and no reduction in lipid peroxidation (MDA) or antioxidant capacity (TEAC)	[96]
Brown et al., 2021	Recreational cyclists	12 mg/day, 7 days, (2 capsules/day)	Oral capsules; astaxanthin (AstaReal^®^)	40 km TT, fat oxidation	↑ fat oxidation, ↑ TT performance	[44]
Tsao et al., 2025	Young active adults	28 mg/day, 4 days	Oral capsules (AstaReal^®^); administered after standardized breakfast	Time-to-exhaustion, CK, MDA	↑ TTE, ↓ CK, ↓ MDA, ↓ lipid peroxidation, TEAC unchanged	[60]
Imai et al., 2018	Trained young athletes	6 mg/day, 4 weeks	Softgel capsules (ASTOTS^®^) taken once daily after breakfast for 4 weeks	Lactate, CK, VO_2_max, AT, performance	↓ Lactate, ↓ CK; no change in VO_2_max, AT, performance	[101]
Saeidi et al., 2023	Obese men + HIFT	20 mg/day, 12 weeks	Oral capsules; administered once daily with breakfast; placebo capsules contained corn starch	Adipokines, metabolic health	↓ CTRP2/9, ↓ GDF8, improved metabolic markers	[10]
Nieman et al., 2023	Endurance athletes	8 mg/day, 4 weeks	Oral capsules; astaxanthin derived from *Haematococcus pluvialis* supplied by Lycored; starch beadlet formulation; ingested once daily with the first meal	Immune-related plasma proteins	Prevents ↓ immune proteins post-exercise	[57]
Muscle Damage and Recovery						
Klinkenberg et al., 2013	Cyclists	20 mg/day, 4 weeks	Oral gelatin capsules; AX extract from *Haematococcus pluvialis* dissolved in sunflower oil, with added vitamin C (60 mg/capsule) and vitamin E (10 mg/capsule); (2 capsules with breakfast and 3 capsules with dinner).	cTnT, MDA, TAC	No effect on troponin or MDA.	[97]
Djordjevic et al., 2012	Elite soccer players	4 mg/day, 90 days	Oral capsules	CK, AST, TAS	↓ exercise-induced superoxide (O2•−), ↓ CK, ↓ AST, ↑ TAS	[99]
Waldman et al., 2023	Resistance-trained	12 mg/day, 4 weeks	Oral supplementation	CK, DOMS, IL-6	No significant effects	[98]
Barker et al., 2023	Resistance-trained	12 mg/day, 4 weeks	Oral capsules	DOMS, performance	↓ DOMS, no performance change	[100]
Elderly Studies						
Liu et al., 2018	Elderly 65–82 y	12 mg/day combined with tocotrienol (10 mg/day) and zinc (6 mg/day), 16 weeks + training	Oral capsules	Strength, CSA, mobility	↑ strength, ↑ CSA, ↑ mobility	[24]
Liu et al., 2021	Elderly	12 mg/day tocotrienol (10 mg/day), and zinc (6 mg/day), + aerobic training	Oral capsules	Fat oxidation, endurance	↑ FATox, ↑ endurance	[52]
Meta-Analyses						
Liu et al., 2024	11 RCTs	8–28 mg/day	Not available (meta-analysis)	Fatigue, aerobic performance	↑ FA oxidation, ↑ aerobic performance	[103]
Hasani et al., 2024	9 RCTs athletes	Variable dose	Not available (meta-analysis)	Performance, TAC	↑ cycling performance, ↑ TAC	[104]

Arrows pointing upward (↑) indicate an increase, while arrows pointing downward (↓) indicate a reduction in the reported key findings.

Overall, the integrated analysis of preclinical models, human trials, and meta-analyses indicates that AX is a promising nutraceutical for supporting exercise-induced adaptations, particularly in untrained individuals, older adults, and populations with metabolic dysfunction. Open questions remain regarding its oral bioavailability, which depends on both formulation and co-ingestion with dietary lipids, optimal dosing strategies, timing relative to training, and the interaction between AX and specific exercise modalities. Future research should also clarify the dose–response window that balances beneficial redox effects without impairing endogenous antioxidant activity. Furthermore, understanding how different formulations modulate astaxanthin’s bioavailability and tissue distribution will be critical to fully translate its mechanistic potential into consistent clinical benefits.

To date, most clinical and preclinical investigations have relied on conventional lipid-based preparations such as softgel capsules or crude *Haematococcus pluvialis* extracts dispersed in vegetable oils. While these formulations offer moderate stability, their low aqueous solubility and oxidative sensitivity limit intestinal absorption and result in variable plasma concentrations, which may explain the modest or inconsistent improvements in performance and recovery observed in several trials.

For instance, the studies by Liu et al. (2018, 2021), Nieman et al. (2023), and Brown et al. (2021) used standard oil-based softgels, reporting moderate benefits in fat oxidation and strength gains [24,44,52,57]. In contrast, work employing emulsified or nanoencapsulated formulations as reported by Tsao et al. (2025) and Wang et al. (2023), showed more pronounced metabolic and functional adaptations, including greater mitochondrial biogenesis, enhanced AMPK activation, and superior redox balance [42,60]. Collectively, these findings underscore that AX’s biological efficacy is closely tied to its formulation, which governs its stability, absorption, and tissue distribution.

Given AX’s central role in mitochondrial health, optimizing its delivery to oxidative tissues such as skeletal muscle and liver remains a crucial step toward maximizing its nutraceutical potential. The next section will therefore examine current formulation and delivery strategies designed to enhance AX stability, bioavailability, and targeted distribution.

## 5. Formulations and Targeted Delivery Strategies

### 5.1. Delivery Strategies to Improve Solubility, Stability and Absorption

The nutraceutical potential of AX is limited by several inherent physicochemical properties, including extremely low aqueous solubility (approximately 0.3–0.5 µg/L), susceptibility to photo-oxidative degradation, and overall poor bioavailability. The preclinical and clinical studies indicate that astaxanthin can modulate exercise adaptation through antioxidant, mitochondrial, and metabolic pathways. However, the variability in outcomes across trials highlights that efficacy is strongly influenced by factors such as dosage, duration, and most critically the formulation and bioavailability of astaxanthin. Across the animal and human studies reviewed in Section 2.1, Section 2.2, Section 2.3, Section 2.4 and Section 2.5, AX was delivered predominantly through lipid-containing matrices, including diet-mixed preparations, oil-based oral suspensions, and softgel capsules. These modalities are consistent with established principles of carotenoid bioavailability, as co-ingestion with dietary fats enhances micellar formation and can increase AX absorption by 2- to 4-fold. Studies employing diet-mixed AX ensured continuous lipid-associated intake, while oral gavage typically used oil-dissolved AX to maximize solubility. Human trials uniformly utilized softgel formulations containing AX in vegetable oils. This methodological consideration is crucial for interpreting AX’s biological effects, as differences in delivery and absorption likely contribute to the variability observed across studies. These features have encouraged the development of numerous delivery strategies aimed at improving the compound’s stability and functional bioactivity. Such approaches enhance molecular protection, improve gastrointestinal transport, and facilitate enterocytic absorption, with reported increases in bioavailability of approximately 2–10 times depending on the delivery system used [1]. A rational selection of delivery strategy, based on the application matrix and physiological objectives, can make a substantial difference in biological efficacy. The main strategies that maximize metabolic and functional effects in exercise models include the use of nanoemulsions, liposomes, SLNs, Nanostructured Lipid Carriers (NLCs), protein complexes or cyclodextrins [105].

Astaxanthin nanoparticles possess distinctive physicochemical and biological properties that derive from the conjugated polyene structure and amphipathic nature of the AX molecule itself. This dual hydrophilic–lipophilic configuration governs nanoparticle morphology, oxidative stability, and cellular uptake. Studies using flash nanoprecipitation and PLGA-based encapsulation have demonstrated that AX-NPs exhibit high encapsulation efficiency (up to 96%), strong photostability, and controlled release kinetics linked to AX’s redox potential rather than to polymeric composition [106,107]. Furthermore, chitosan- and protein-based AX-nanoparticles enhance antioxidant performance and cellular bioavailability through electrostatic interactions and hydrogen bonding between AX’s keto–hydroxyl groups and matrix amino residues [108]. Carrier-free AX nanoparticles also display unique self-assembly behavior, forming amorphous nanostructures with superior stability and delayed release profiles [109]. These findings underscore that the physicochemical identity of AX fundamentally determines nanoparticle performance, distinguishing AX-nanoparticles from conventional nanocarriers that rely solely on polymeric or lipidic stabilization. Similar relationships between molecular structure and nanocarrier functionality have been described for anthocyanin-loaded nanoparticles, where the conjugated polyphenolic structure governs particle morphology, stability, and biological performance [110].

#### 5.1.1. Nanoemulsions and Microemulsions

Nanoemulsions are among the most widely studied systems for improving the dispersibility of AX in food matrices and physiological environments. These formulations, typically exhibiting droplet sizes of 30–200 nm, provide a large interfacial area and enhanced oxidative stability through the use of selected food-grade surfactants and carrier oils [111]. Animal and human studies have demonstrated increases in solubility and oral bioavailability of approximately 3 to 6 times compared with non-encapsulated AX. In addition, nanoemulsions promote more efficient intestinal absorption and offer substantial protection against oxidative degradation, up to 40–60%, by limiting the exposure of AX to the external environment [112,113]. Microemulsions, which are characterized by droplet sizes below 50 nm and differ from nanoemulsions in thermodynamic stability and composition, exhibit comparable functional benefits. They enable solubilisation levels up to 100–300 times higher than in pure water, making them valuable for industrial applications, although they generally require higher surfactant concentrations [114,115].

#### 5.1.2. Liposomes

Liposomes, ranging in size from 80 to 300 nm, provide a lipid microenvironment that protects AX from oxidation and photodegradation. The use of natural phospholipids improves light stability by up to 50 per cent and increases intestinal absorption by approximately 1.5 and 3 times compared to the free form [116,117]. In addition, the phospholipid matrix facilitates interaction with biological membranes and can increase transcellular transport. In fact, the encapsulation of astaxanthin within phospholipid bilayers mimics its natural orientation in biological membranes, positioning the hydrophobic polyene chain within the lipid core and exposing terminal hydroxyls toward aqueous interfaces [118]. Recent studies show significantly slower release kinetics compared to the free molecule, with cumulative values rarely exceeding 30–40% even after 24–48 h, confirming prolonged and controlled release [119,120]. Thanks to their compatibility with dietary phospholipids or plant derivatives, liposomes represent a promising platform for nutraceutical and clinical applications focused on muscle metabolism and weight control. Improved bioavailability achieved through nanoemulsion or liposomal systems directly enhances AX delivery to mitochondria-rich tissues such as skeletal muscle and liver, where it exerts its AMPK- and PGC-1α-mediated metabolic effects. This mechanistic link between formulation efficiency and metabolic efficacy highlights the importance of optimizing AX delivery systems for translational applications in exercise physiology and obesity management.

#### 5.1.3. Solid Lipid Nanoparticles (SLNs) and Nanostructured Lipid Carriers (NLCs)

SLNs typically range from 100 to 250 nm in size and exhibit encapsulation efficiencies of 50 and 80%. These systems provide a solid lipid matrix that entraps and protects AX, enhancing its chemical stability and shelf-life. However, high crystallinity can limit the load and promote the expulsion of the compound during solidification, with a loss of 10–20% [121,122]. NLCs partially solve this problem by combining solid and liquid lipids, producing a less orderly network capable of increasing encapsulation capacity to 80–95%. In vivo studies report higher AX bioavailability, approximately 4 to 7 times increases, and improved stability during digestion, with gains of up to 60% compared with SLNs [123,124,125]. Both systems show high gastrointestinal absorption, protection against the acidic environment of the stomach and improved tissue distribution [124,125], which are important aspects for applications targeting muscle function and lipid metabolism regulation.

Beyond these physicochemical parameters, the protective performance of lipid nanoparticles is strongly influenced by AX’s molecular properties. SLNs and NLCs encapsulating AX provide a hydrophobic lipid matrix that restricts oxygen diffusion, protecting the conjugated polyene structure from oxidative degradation

The encapsulation efficiency and release kinetics depend strongly on AX’s lipophilicity and redox potential, showing higher stabilization in saturated lipid cores [106].

#### 5.1.4. Protein- or Cyclodextrin-Based Complexes

Encapsulation using protein complexes (whey, casein, zein) with diameters ranging from 50 to 200 nm creates hydrophobic interactions and non-covalent bonds, which improve the stability and dispersibility of AX in water and increase its solubility up to 20 times. These complexes have demonstrated 30–50% greater resistance to digestive degradation, along with intestinal absorption improvements of approximately 2 to 4 time [126,127,128]. Cyclodextrins, particularly β-cyclodextrin, form complexes that can stabilize carotenoids and increase their solubility by up to 100–500 times, with encapsulation efficiency typically ranging from 40 to 70%. In addition, studies report improved bioavailability and a 50–80% reduction in oxidative degradation rates [129,130].

### 5.2. Linking Delivery Efficiency to Biological Function

The relationship between the delivery system and the biological function of AX has become a central topic in nutraceutical research. The lipophilic nature of AX limits its efficient entry into the enterocyte compartment and subsequent distribution in metabolically active tissues. For this reason, the concept of delivery to function has been developed, which directly links the physicochemical design of the transport system to its physiological efficacy. Based on this, a specifically formulated system allows AX to reach effective concentrations in areas where it can directly modulate oxidative stress, energy metabolism and muscle function. In addition, the optimal delivery system also improves its application in the food and nutraceutical fields. Nanoemulsions and NLCs with sizes smaller than 200 nm, facilitate absorption through mechanisms mediated by chylomicrons and increase entry into the lymphatic circulation. This leads to a higher concentration of carotenoids in tissues with high oxidative turnover, such as skeletal muscle and liver, with tissue increases of up to 5 times compared to the free form [112,123]. More efficient biodistribution increases the possibility of AX interacting with mitochondrial membranes and proteins that regulate cellular metabolism. Encapsulation in liposomes and NLCs improves the incorporation of AX into lipid bilayers due to the enhanced compatibility between the carrier matrix and biological phospholipids. Comparative studies show that liposomal formulations increase inhibition of lipid peroxidation by 40–70% compared with non-encapsulated AX [116]. Protein complexes also exhibit superior effects in reducing oxidative markers due to enhanced digestive stability and a more gradual release, which prolongs systemic exposure [126]. The improvement in bioavailability achieved with nanoemulsions, NLCs and liposomes amplifies AX’s ability to modulate key pathways related to mitochondrial biogenesis, anti-inflammatory capacity and the management of exercise-induced muscle damage. Studies in mouse and human models show that improved formulations lead to greater activation of PGC-1α, AMPK and SIRT1, with increases in exercise endurance and reductions in post-exercise oxidative damage of up to 30–40% compared to free AX [42]. The effect depends on the form of delivery, confirming that structural design directly influences the physiological response. Highly efficient encapsulation formulations, such as NLCs and low-surfactant nanoemulsions, have shown superior ability to modulate lipolysis, insulin sensitivity and fatty acid oxidation. This results from greater stability under digestive conditions and higher carotenoid concentration in metabolically active tissues. In studies evaluating obesity-related parameters, these formulations lead to a 20–35% reduction in plasma triglycerides and visceral adipose tissue, a result significantly higher than that obtained with non-encapsulated AX [124,131]. Table 3 summaries the delivery systems used to improve the solubility, stability and absorption of AX. The advantages and possible applications in the food and nutraceutical fields are reported for each system.

### 5.3. Astaxanthin-Mitochondria Interactions

The interactions of AX with the mitochondrial compartment are key to understanding its effects on muscle metabolism, exercise adaptation and energy regulation. Once absorbed, AX inserts into the phospholipid bilayers of mitochondrial membranes. Integration is facilitated by its polar conformation at the ends and lipophilic conformation at the center, which allows the carotenoid to occupy a transverse position in the bilayer. Cellular and animal models show that encapsulated AX protects mitochondrial phospholipids, such as cardiolipin, from degradation with 40–70% greater efficacy than the free form, thanks to greater intracellular stability and mitochondrial permanence [5,132]. In addition, mitochondrial interactions include increased ATP availability and reduced energy cost during exercise. Studies conducted with nanoemulsified forms show increases in muscle ATP production of up to 15–25% and a reduction in post-exercise oxidative damage of 30–40% [111]. Ambati et al. (2014) [1] show that incorporation into mitochondria reduces excessive membrane fluidity and can limit ROS production by 30–50% and preserve mitochondrial membrane potential. This function is particularly relevant in muscle during exercise, where increased electron flow makes mitochondrial membranes vulnerable to oxidative damage. Formulations capable of promoting the accumulation of AX in mitochondria can enhance both the structural protection of internal membranes and the efficiency of bioenergetic pathways. Carrier proteins, such as modified serum derivatives, can increase the transfer of AX to mitochondria, improving its intracellular stability and antioxidant function. In mouse models, these complexes increased the oxidation of lipid substrates in muscle and improved exercise endurance [132]. Liposomal formulations associated with molecules such as triphenylphosphonium (TPP+) showed up to 5-fold increases in mitochondrial transport compared to conventional liposomes and greater protection from oxidative stress in differentiated muscle models [124]. Positively charged lipid nanocarriers (NLCs and SLNs) improve interaction with internal membranes, promoting release closer to the respiratory chain complexes. These systems have shown increased protection of complexes I and III and a 20–30% increase in oxidative capacity in cellular models [124,133].

Furthermore, formulations such as liposomes and NLCs show a reduction in mitochondrial ROS of up to 30–60% compared to non-encapsulated forms [124,133]. This protection contributes to the maintenance of membrane potential and reduces mitochondrial dysfunction induced by intense exercise or metabolic stress conditions. AX also influences mitochondrial biogenesis through the activation of signaling pathways. The increased bioavailability achieved through nanoemulsions and NLCs makes this modulation more evident, with increases in PGC-1α expression of between 20% and 50%. Activation of these pathways promotes higher mitochondrial density and oxidative capacity in skeletal muscle, adaptations essential for endurance performance and improved lipid metabolism [111]. From a metabolic point of view, targeted mitochondrial delivery also amplifies AX’s benefits for body-weight regulation. By improving the ability of muscle and liver cells to oxidize lipids, advanced formulations contribute to reductions in plasma triglycerides, enhanced insulin sensitivity, and decreased fat accumulation. Preclinical studies using optimized delivery systems have reported reductions of 20–35% in visceral adipose tissue compared with administration of the non-encapsulated compound [124,131].

## 6. Future Directions and Conclusions

This review provides a critical overview of the current evidence regarding the nutraceutical potential of AX in muscle metabolism, exercise adaptation, and obesity management. AX emerges as a multifunctional nutraceutical, modulating metabolic, inflammatory, and mitochondrial pathways in skeletal muscle, adipose tissue, and liver. Evidence in muscle metabolism consistently highlights AX’s ability to support mitochondrial function, enhance fatty acid oxidation, and preserve muscle integrity under metabolic or mechanical stress. In exercise adaptation, AX demonstrates benefits in oxidative balance, endurance, and recovery, although human data remain heterogeneous. In obesity, AX contributes to improved adipose tissue remodeling, hepatic lipid handling, and systemic metabolic flexibility.

Although preclinical evidence is robust, clinical translation presents inconsistencies in trial design, formulation, and bioavailability. To address these gaps, integrated multi-omic approaches, such as metabolomics, lipidomics, and proteomics, are needed to identify biomarkers and map metabolic interactions along the liver–adipose–muscle axis [134,135].

Future perspectives should emphasize the translational potential of AX in clinical settings. Based on current evidence, AX represents a promising adjuvant nutraceutical in populations with metabolic inflexibility, including individuals with obesity, insulin resistance, and age-related muscle decline. Future randomized controlled clinical trials should prioritize combined interventions integrating AX supplementation with structured exercise programs, given the consistent synergistic effects observed on mitochondrial function, lipid oxidation, and muscle performance. Trials should adopt standardized dosing, adequate intervention duration, and clinically relevant endpoints, including metabolic flexibility, insulin sensitivity, muscle strength, recovery biomarkers, and adipokine profiles. Moreover, advances in formulation technologies, such as encapsulation and mitochondria-targeted delivery systems, should be directly tested in clinical trials to determine whether improved bioavailability enhances therapeutic efficacy.

In summary, AX demonstrates significant potential to improve muscle metabolism, optimize exercise-induced adaptations, and mitigate obesity-related dysfunction. Continued innovation in delivery systems and omics-guided research will be critical to integrating AX into precision nutrition and metabolic health strategies.

## Figures and Tables

**Table 3:** Summary diagram of astaxanthin delivery systems, the main advantages and food and nutraceutical applications.

Delivery System	Advantages	Food Applications	Nutraceutical Applications	References
Nanoemulsions	Increased solubility and bioavailability; improved oxidative protection; good dispersion in aqueous matrices	Functional drinks, yogurt, juices	Softgels, liquid sachets	[112,113]
Microemulsions	Spontaneous formation; high thermodynamic stability; excellent solubilisation	Concentrated sports drinks, liquid emulsions	High solubility liquid formulations	[114,115]
Liposomes	Protection against photo-oxidative degradation; good affinity with cell membranes; controlled release	Limited food applications (high cost)	Softgels, fast-absorbing capsules	[116,117]
SLNs	High stability; good carotenoid protection; improved absorption	Experimental functional snacks, enriched powders	Capsules, extended-release tablets	[121,122]
NLCs	Higher loading capacity than SLNs; superior bioavailability; better stability during digestion	Fortified dry foods, sports mixes	Powders and tablets for lipid metabolism	[123,124,125]
Protein complexes	Greater solubility and digestive resistance; gradual release; use of safe food matrices	Protein yogurt drinks	Protein powder supplements	[126,127,128]
Cyclodextrin complexes	Increase apparent solubility; strong protection against oxidation; better thermal stability	Instant drinks, soluble powders	Orodispersible tablets, single-dose sachets	[129,130]

## Data Availability

Data are available from the authors upon reasonable request.

## Abbreviations

The following abbreviations are used in this manuscript:

4-HNE	4-hydroxy-2-nonenal
8-OHdG	8-hydroxy-2′-deoxyguanosine
ACC	Acetyl-CoA carboxylase
AMPK	AMP-activated protein kinase
aP2	Adipocyte protein 2
AKT	Protein kinase B
ARA	Arachidonic acid
AST	Aspartate aminotransferase
ATP	Adenosine triphosphate
AX	Astaxanthin
C/F	Capillary-to-fiber ratio
CAF	Capillaries per fiber
CD36	Cluster of differentiation 36
CK	Creatine kinase
COX-2	Cyclooxygenase-2
CPT1	Carnitine palmitoyltransferase 1
CSA	Cross-sectional area
cTnT	Cardiac troponin T
CTRP2	C1q/TNF-related protein 2
CTRP9	C1q/TNF-related protein 9
CYP27A1	Cytochrome P450 27A1
CYP7A1	Cholesterol 7α-hydroxylase
DHA	Docosahexaenoic acid
DOMS	Delayed onset muscle soreness
EPA	Eicosapentaenoic acid
FAS	Fatty acid synthase
FCSA	Muscle fiber cross-sectional area
GDF8	Growth differentiation factor 8
GDF15	Growth differentiation factor 15
GLUT4	Glucose transporter type 4
GPDH	Glycerol-3-phosphate dehydrogenase
HFD	High-fat diets
Hif-2α	Hypoxia-inducible factor 2α
HIIT	High-Intensity Interval Training
IL	Interleukin
iNOS	Inducible nitric oxide synthase
IRS-1	Insulin receptor substrate-1
JNK	C-jun *N*-terminal kinase
LDH	Lactate dehydrogenase
Ldlr^−^/^−^	Low-density lipoprotein receptor–deficient
LXRα	Liver X receptor alpha
MAPK	Mitogen-activated protein kinase
MDA	Malondialdehyde
MVC	Maximum voluntary contraction
NAD^+^	Nicotinamide adenine dinucleotide
NF-κB	Nuclear factor kappa B
NLCs	Nanostructured Lipid Carriers
Nrf1	Nuclear respiratory factor 1
Nrf2	Nuclear factor erythroid 2-related factor 2
PGC-1α	Peroxisome proliferator-activated receptor gamma coactivator 1-alpha
PI3K	Phosphoinositide 3-kinase
PPARα	Peroxisome proliferator-activated receptor alpha
PPARγ	Peroxisome proliferator-activated receptor gamma
PPARδ	peroxisome proliferator-activated receptor delta
PRDM16	PR/SET domain containing 16
PUFA	Polyunsaturated fatty acid
RCTs	Randomized controlled trial
RONS	Reactive oxygen and nitrogen species
ROS	Reactive oxygen species
SCD-1	Stearoyl-CoA desaturase-1
SIRT1	Sirtuin 1
SIRT3	Sirtuin 3
SLNs	Solid lipid nanoparticles
SREBP-1c	Sterol regulatory element-binding protein-1c
TFAM	Mitochondrial transcription factor A
TNF-α	Tumor necrosis factor-alpha
TPP+	Triphenylphosphonium
UCP1	Uncoupling protein 1
WAT	White adipose tissue
